# Cutaneous Multiple Myeloma

**DOI:** 10.7759/cureus.17779

**Published:** 2021-09-06

**Authors:** Leela Krishna Vamsee Miriyala, Deepti Avasthi

**Affiliations:** 1 Internal Medicine, St. Vincent Mercy Medical Center, Toledo, USA

**Keywords:** chemotherapy-induced, extramedullary multiple myeloma, amyloidosis and multiple myeloma, secondary primary cancer, cutaneous multiple myeloma, cutaneous malignant melanoma, lenalidomide, squamous cell carcinoma (scc)

## Abstract

Multiple myeloma is a plasma cell dyscrasia characterized by abnormal bone marrow clonal plasma cells, histological confirmation of plasmacytoma, monoclonal protein in serum or urine, and evidence of end-organ damage. Organ involvement in multiple myeloma manifests as CRAB (hyperCalcemia, Renal insufficiency, Anemia, lytic Bone lesions). Cutaneous complications in multiple myeloma have been reported in many different phenotypes such as cryoglobulinemia rash, bruising, amyloid deposition, and squamous cell carcinoma. However, cutaneous metastasis of multiple myeloma is very rare with fewer than 100 cases described in the literature so far. Here, we present a case of biopsy-confirmed primary cutaneous multiple myeloma. Our case has other less common features of multiple myeloma such as renal amyloidosis and a coexisting malignant melanoma. This case report describes a unique presentation of multiple myeloma to understand the disease better.

## Introduction

Multiple myeloma is uncommon cancer. Cutaneous metastasis in multiple myeloma is rarer with fewer than 100 cases described in the literature so far. Cutaneous metastasis in multiple myeloma typically occurs in late-stage disease and represents a poor prognosis with an approximate median survival of eight months.

Here, we report the case of a 61-year-old male patient with multiple myeloma, subtype lambda light chain, who developed skin metastasis during the course of the disease. Despite aggressive chemotherapy, he rapidly declined and succumbed to the disease within eight weeks of skin involvement. Furthermore, the rarity of this case is that he also developed invasive malignant melanoma after a year of multiple myeloma diagnosis. The development of secondary primary cancers is a known phenomenon. However, the association of malignant melanoma in a multiple myeloma patient is not well established in the literature. This is a unique case of multiple myeloma complicated by cutaneous metastasis and the development of malignant melanoma, which has never been reported to date. This case report could be the opener for future studies on exploring the risk/association of primary skin cancers in patients with multiple myeloma with skin metastasis.

## Case presentation

A 61-year-old male who had initially presented to the emergency room in October 2018 with complaints of exertional dyspnea and left-sided anginal chest pain for four to five weeks was eventually admitted for the management of acute-on-chronic systolic congestive heart failure and hypertensive urgency. His medical history was significant for essential hypertension, coronary artery disease status post-percutaneous coronary intervention twice with a total of three stent placements (one in right coronary artery in August 2014, two in left anterior descending artery, and obtuse marginal artery in October 2014). Family history was unremarkable for any cancers. His social history was also insignificant. He was found to have ischemic cardiomyopathy with a left ventricular ejection fraction of 20% with global hypokinesis on echocardiography in October 2018. He underwent a coronary angiogram which demonstrated significant occlusion in the left anterior descending and large diagonal artery requiring three more stent placements.

During that admission, the patient was also found to have acute kidney injury with a creatinine level of 1.7 mg/dL and a glomerular filtration rate of 40. Complete blood count and basic metabolic panel revealed no significant abnormalities (Table 1). Further workup revealed nephrotic range proteinuria (Table 1). Workup for infectious, autoimmune, and obstructive etiologies was negative (Table 2). Pan hypogammaglobulinemia was identified and beta-2 microglobulin levels were elevated (3.6 mg/dL) (Table 2). Protein electrophoresis identified a paraprotein band and a large amount of free lambda light chains, with a very low kappa/lambda ratio in both serum and urine (Table 3). No anemia or hypercalcemia was noted. The patient subsequently underwent a bone marrow biopsy which demonstrated 30-40% monoclonal lambda (+) plasma cells and negative flow cytometric immunophenotyping, which was compatible with plasma cell myeloma (multiple myeloma). Due to worsening renal function and rising urine light chains, he underwent a left renal biopsy as well which revealed amyloidosis-lambda light chain renal amyloidosis.

**Table 1:** Complete blood count with differential, basic metabolic panel, and urine chemistry. WBC: white blood cell; RBC: red blood cell; MCV: mean corpuscular volume; MCH: mean corpuscular hemoglobin; MCHC: mean corpuscular hemoglobin concentration; RDW: RBC distribution width; BUN: blood urea nitrogen; CO_2_: carbon dioxide (represents blood bicarbonate levels); GFR: glomerular filtration rate; UA: urine analysis

Complete blood count with differential			Basic metabolic panel			Urine chemistry	
WBC	14.5 (High)	3.5–11.3 k/uL	Glucose	114 (High)	70–99 mg/dL	Glucose, Urine	Negative
RBC	4.71	4.21–5.77 m/uL	BUN	18	8–23 mg/dL	Bilirubin, Urine	Negative
Hemoglobin	14.6	13.0–17.0 g/dL	Creatinine	1.70 (High)	0.70–1.20 mg/dL	Ketones, Urine	Negative
Hematocrit	43.7	40.7–50.3 %	Calcium	8.8	8.6–10.4 mg/dL	Specific gravity, UA	1.022
MCV	92.8	82.6–102.9 fL	Sodium	141	135–144 mmol/L	Urine Hgb	Small
MCH	31.0	25.2–33.5 pg	Potassium	4.3	3.7–5.3 mmol/L	pH, UA	5.0
MCHC	33.4	28.4–34.8 g/dL	Chloride	105	98–107 mmol/L	Protein, UA	3+
RDW	12.3	11.8–14.4 %	CO_2_	25	20–31 mmol/L	Urobilinogen, Urine	Normal
Platelets	340	138–453 k/uL	Anion gap	11	9–17 mmol/L	Nitrite, Urine	Negative
Segmented neutrophils	73 (High)	36–65%	GFR Non-African American	41 (Low)	>60 mL/min	Leukocyte esterase, Urine	Negative
Lymphocytes	18 (Low)	24–43 %	GFR African American	50 (Low)	>60 mL/min	Total protein, Urine	497
Monocytes	6	3–12%				Creatinine, Urine	61.2
Eosinophils	2	1–4%				Urine protein creatinine ratio	8.12
Basophils	1	0–2%					
Immature granulocytes	0	0%					
Segmented absolute	10.59 (High)	1.50–8.10 k/uL					

**Table 2:** Findings of serum protein electrophoresis with immunofixation, serum free light chain assay, urine protein electrophoresis and immunofixation, and urine free light chain assay.

Serum protein electrophoresis with immunofixation			Serum free light chain assay			Urine protein electrophoresis with immunofixation		Urine free light chain assay		
Total protein	4.1 (Low)	6.4–8.3 g/dL	Kappa free light chains	0.67	0.37–1.94 mg/dL	Total protein	460 (High)	Kappa free light chains	5.35 (Low)	0.14–2.42 mg/dL
Albumin (calculated)	2.4 (Low)	3.2–5.2 g/dL	Lambda free light chains	2055.70 (High)	0.57–2.63 mg/dL	Albumin	Detected	Lambda free light chains	402 (High)	0.02–0.67 mg/dL
Albumin	58	45–65%	Free kappa/lambda ratio	0.00 (Very low)	0.26–1.65	Alpha-1 globulin	Detected	Free kappa/lambda ratio	0.01 (Very low)	2.04–10.37
Alpha-1-globulin	0.3	0.1–0.4 g/dL				Alpha-2 globulin	Detected			
Alpha 1	7 (High)	3–6%				Beta globulin	Detected			
Alpha-2-globulin	0.7	0.5–0.9 g/dL				Gamma globulin	Detected			
Alpha 2	18 (High)	6–13 %				Urine immunofixation: Urine positive for monoclonal free lambda light chains (Bence Jones proteins). Quantification: 35 mg/dL				
Beta globulin	0.5	0.5–1.1 g/dL								
Beta	12	11–19%								
Gamma globulin	0.2 (Low)	0.5–1.5 g/dL								
Gamma globulin	5 (Low)	9–20%								
Total protein Sum	4.1 (Low)	6.3–8.2 g/dL								
Total Protein Sum (%)	100	98–102%								
Serum immunofixation: A zone of restriction is present in the gammaglobulin region. Confirmed by immunofixation to be monoclonal lambda free chains. Quantification: 0.08 g/dL										

**Table 3:** Findings of serology, immunoglobulin panel, and hepatitis panel. ANA: antinuclear antibodies; ANCA: antineutrophil cytoplasmic antibodies; C: complement; Ig: immunoglobulin; Ab: antibody

Serology			Immunoglobulin panel			Hepatitis panel	
ANA	Negative		IgG	<300 (Low)	700–1,600 mg/dL	Hepatitis B surface Ag	Nonreactive
C3	130	90–180 mg/dL	IgA	15 (Low)	70–400 mg/dL	Hepatitis B core Ab	Nonreactive
C4	40	10–40 mg/dL	IgM	<25 (Low)	40–230 mg/dL	Hepatitis C Ab	Nonreactive
ANCA myeloperoxidase	3	<100 AU/mL				Hepatitis A Ab IgM	Nonreactive
ANCA proteinase 3	7	<100 AU/mL	Beta-2 microglobulin	3.6 mg/dL	0.7–1.8 mg/dL		

He was started on first-line therapy for multiple myeloma with pomalidomide, lenalidomide, and dexamethasone in June 2019. Over the following year, his paraproteinemia profile transiently improved. Eventually, he developed progressive renal failure requiring initiation of renal replacement therapy with hemodialysis in June 2020. Moreover, his urine free lambda light chains had rapidly increased. Lack of response to chemotherapy prompted the switch from lenalidomide to daratumumab in June 2020, and therapy was continued with daratumumab, pomalidomide, and dexamethasone.

**Figure 1:**
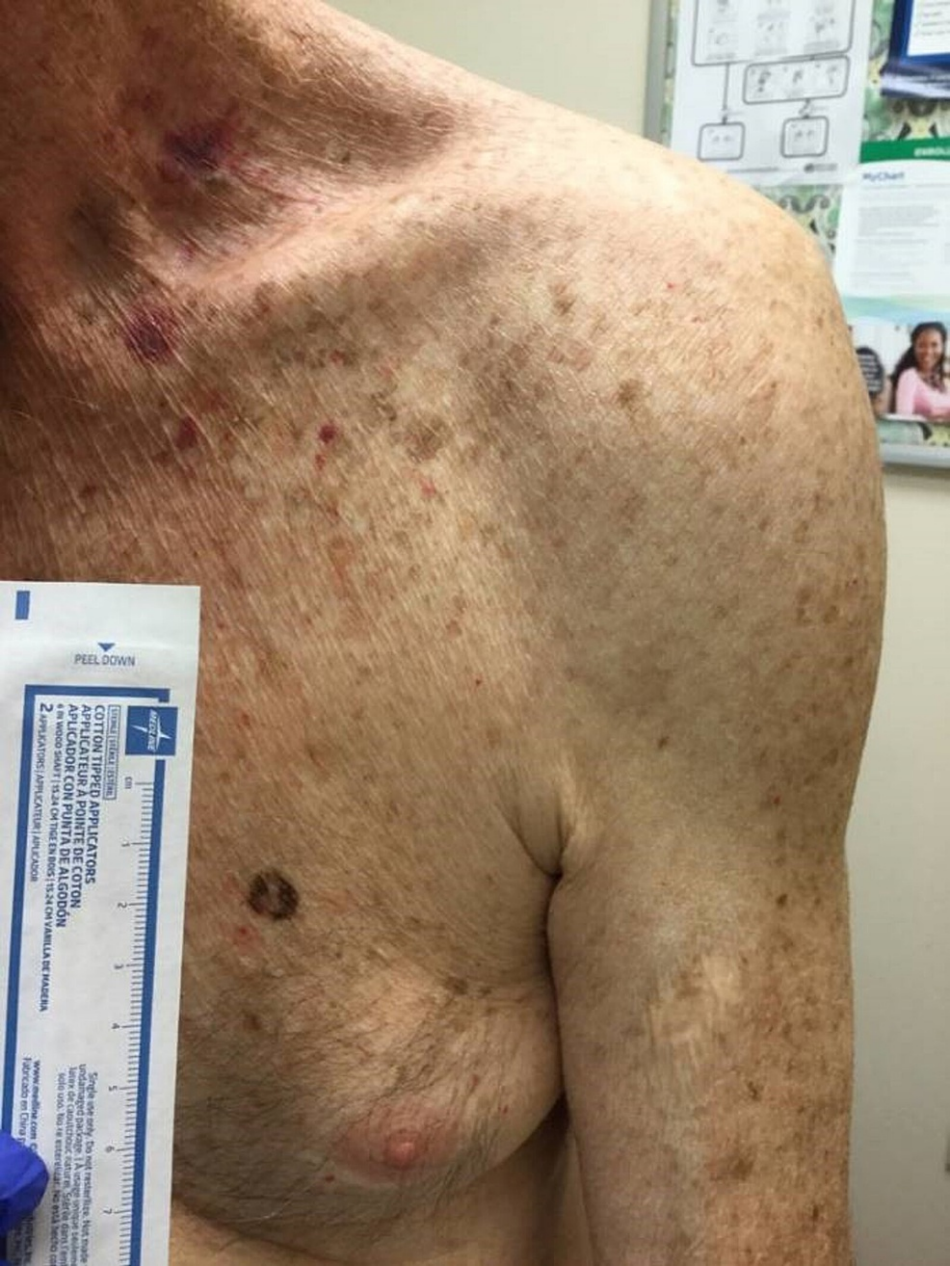
A 9 × 9 mm lesion with heterogenous pigmentation over the left anterior chest, indicative of melanoma in situ.

**Figure 2:**
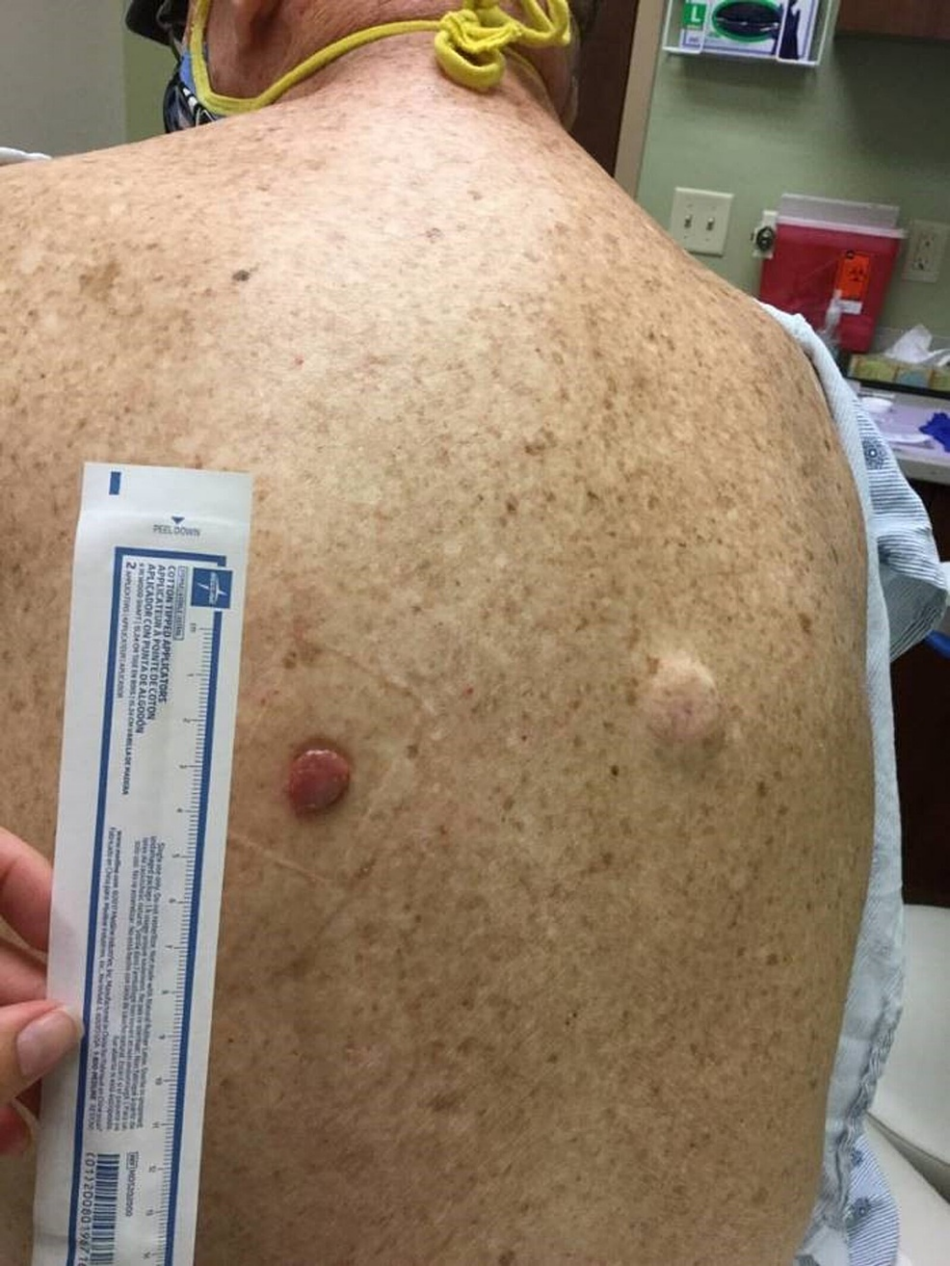
A 15 × 12 mm raised growth over the left mid-back, indicative of invasive malignant melanoma.

**Figure 3:**
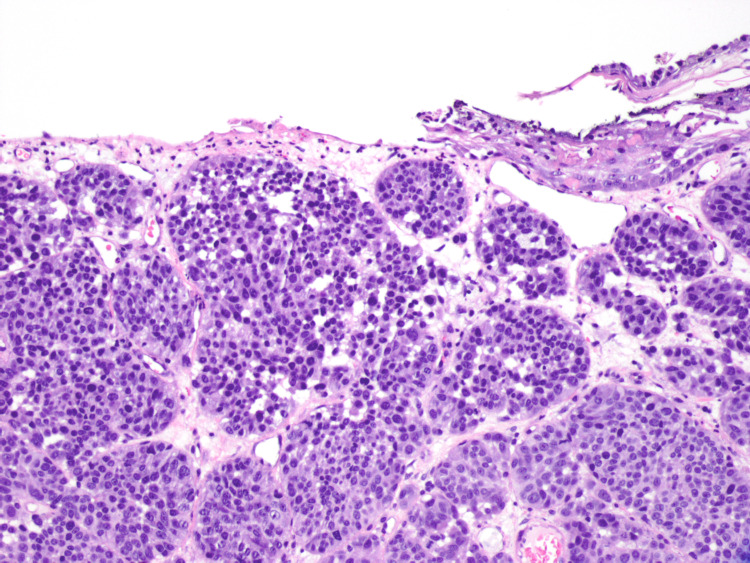
Histopathological image of ulcerated nodular melanoma, left mid-back (10× objective).

**Figure 4:**
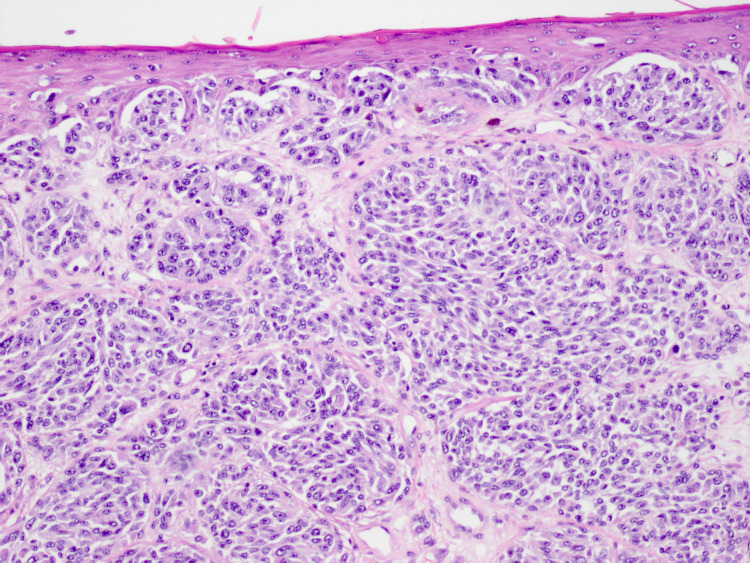
Histopathological image of melanoma in situ in epidermis overlying invasive melanoma in the dermis, left mid-back (10× objective).

**Figure 5:**
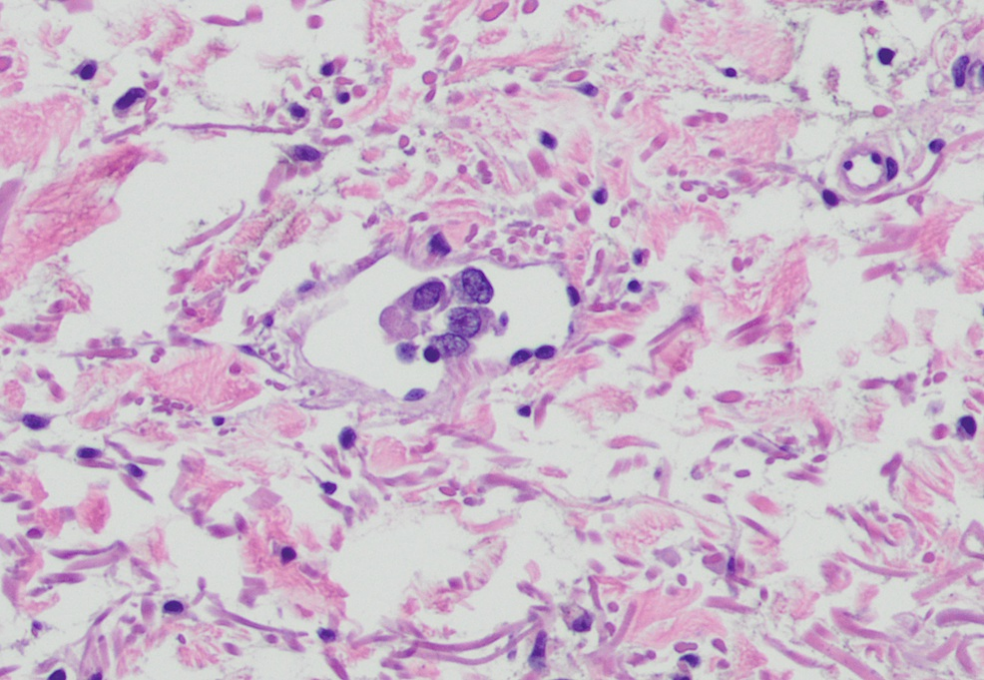
Histopathological image of invasive melanoma with lymphovascular invasion, left mid-back (40× objective).

In August 2020, he complained of new skin growths on his left chest and left mid-back (Figures 1, 2). Shave biopsies were obtained and histopathology demonstrated findings consistent with early nonulcerated melanoma in-situ with pathological stage, pTis of the left anterior chest, and ulcerated invasive nodular malignant melanoma with lymphovascular invasion with pathological stage, pT4b of the left mid-back (Figures 3-5).

**Figure 6:**
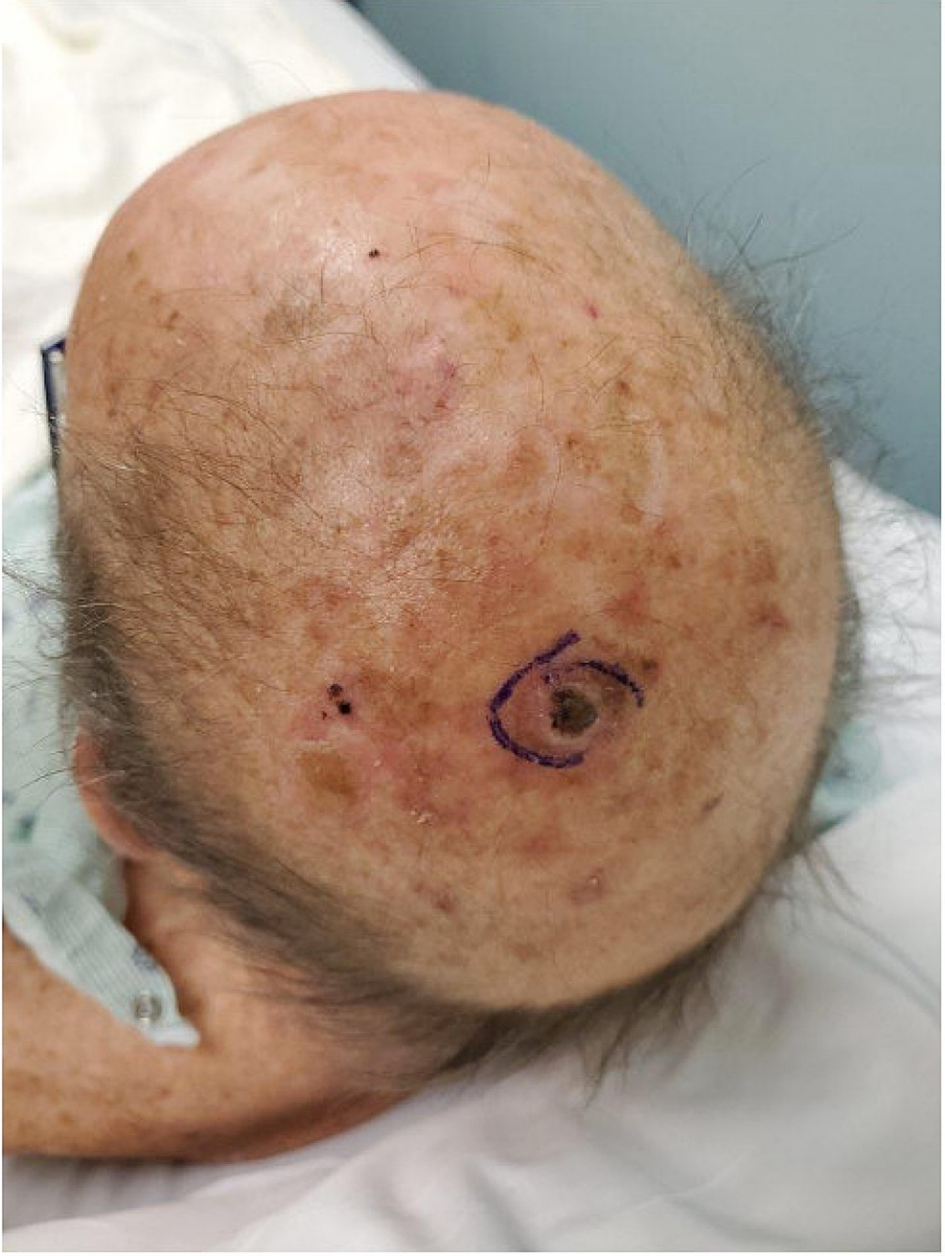
Squamous cell carcinoma of the posterior scalp.

**Figure 7:**
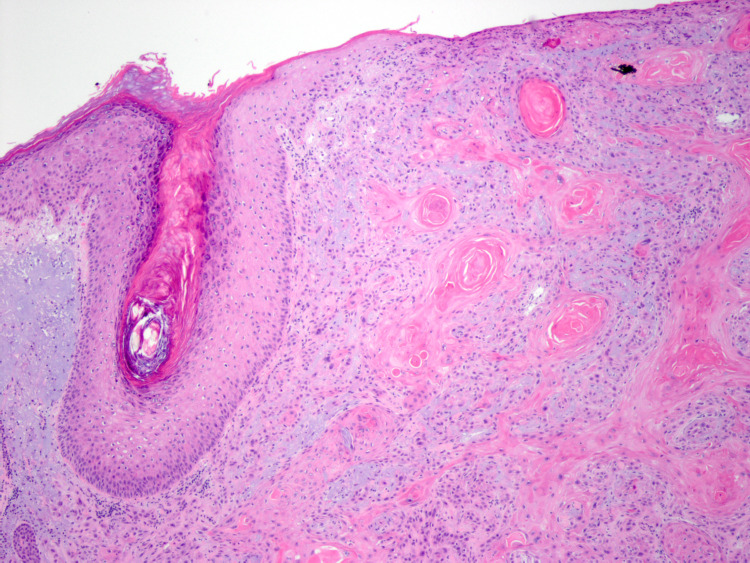
Histopathological image of invasive squamous cell carcinoma, posterior scalp (4× objective).

**Figure 8:**
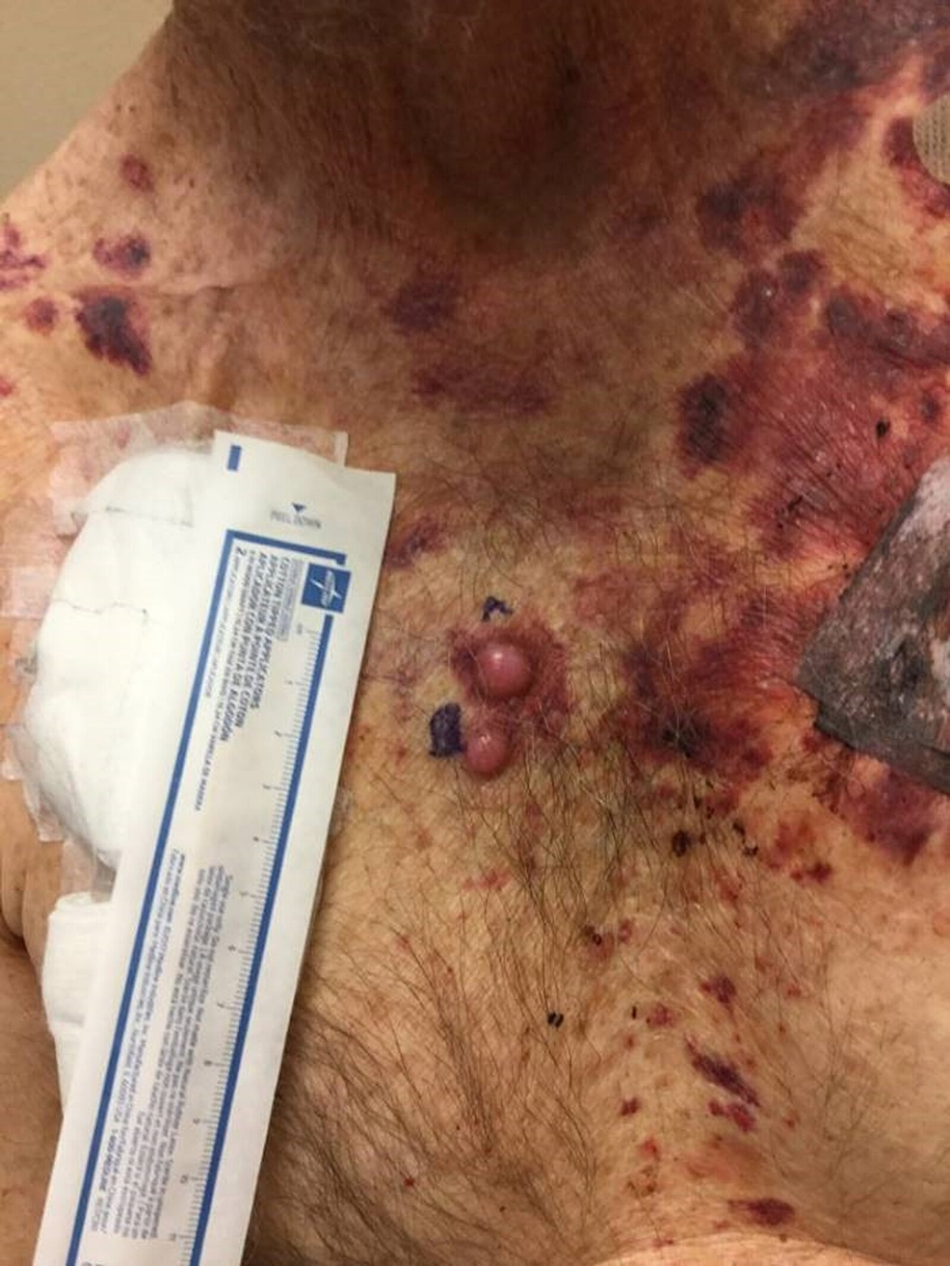
Two raised dome-shaped pinkish nodules measuring 11 × 9 × 3 mm over the right anterior chest, indicative of cutaneous multiple myeloma.

**Figure 9:**
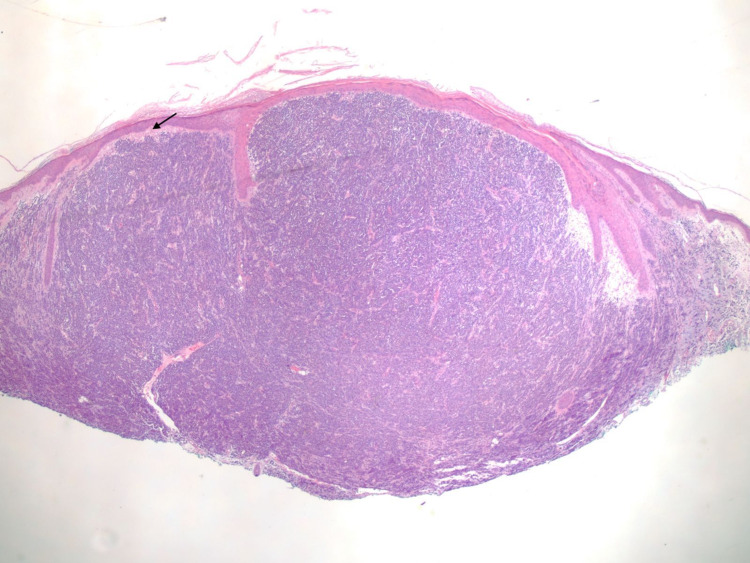
Histopathological image of plasma cell neoplasm, right chest (1× objective). Dome-shaped lesion consisting of dense sheet-like growth of plasmacytoid cells with a small Grenz zone (black arrow).

**Figure 10:**
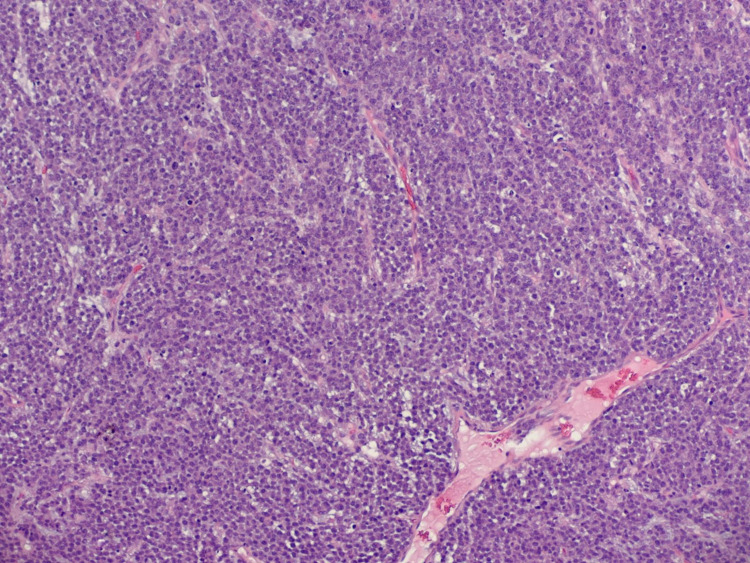
Histopathological image of plasma cell neoplasm, right chest (10× objective). The cell infiltrate shows eccentric nuclei with coarse chromatin and prominent nucleoli.

**Figure 11:**
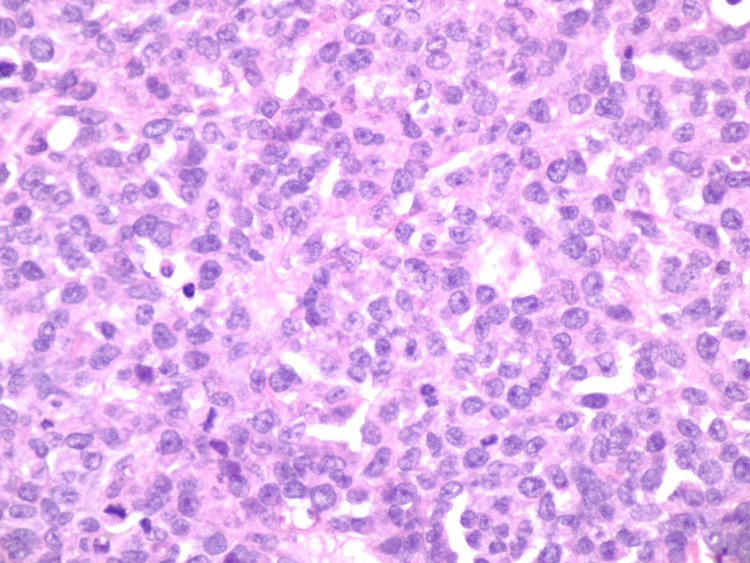
Histopathological image of plasma cell neoplasm, right chest (40× objective). The cell infiltrate shows eccentric nuclei with coarse chromatin and prominent nucleoli. Scattered mitotic figures are present.

**Figure 12:**
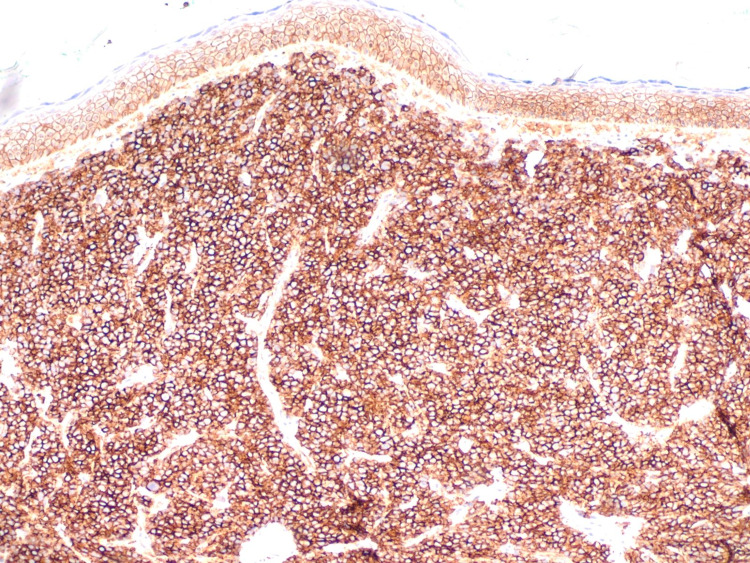
Immunohistochemistry of cutaneous plasmacytoma/multiple myeloma. Immunohistochemistry of the infiltrate was strongly positive for CD138 and showed lambda restriction. The infiltrate was negative for kappa, S-100, SOX-10, and melan-A.

In October 2020, he developed a new posterior scalp lesion (Figure 6). He subsequently underwent excision of all three skin lesions along with sentinel lymph node dissection, revealing squamous cell carcinoma of the scalp (Figure 7) and confirmed invasive malignant melanoma. In November 2020, two new pink-colored raised dome-shaped nodules were identified over the right anterior chest (Figure 8). Shave biopsies were obtained and histopathology demonstrated dense sheet-like growth of plasmacytoid cells with a small Grenz zone. The cell infiltrate showed eccentric nuclei with coarse chromatin and prominent nucleoli. Immunohistochemistry of the infiltrate was strongly positive for CD138 and showed lambda restriction, but negative for kappa, S-100, SOX-10, and melan-A (Figures 9-12). These findings were consistent with cutaneous multiple myeloma. The patient was continued with the ongoing treatment with daratumumab, pomalidomide, and dexamethasone. Despite aggressive ongoing chemotherapy, he progressively worsened and eventually deceased within few months of the diagnosis of cutaneous multiple myeloma.

## Discussion

Multiple myeloma is a relatively uncommon cancer accounting for approximately 1-2% of all cancers and slightly more than 18% of hematologic malignancies [1]. The incidence of myeloma is around 7 per 100,000 individuals per year, and the mortality rate is 3.2 per 100,000 individuals per year. In 2017, an estimated 140,779 people were living with myeloma in the United States [2].

Cutaneous multiple myeloma

Multiple myeloma is a malignant plasma cell disorder characterized by neoplastic proliferation of plasma cells producing a monoclonal immunoglobulin. The plasma cells proliferate in the bone marrow and can result in extensive skeletal destruction with osteolytic lesions, osteopenia, and/or pathological fractures. In addition, multiple myeloma can manifest with extramedullary involvement, where the neoplastic plasma cells infiltrate organs outside the bone marrow. Approximately 4% of multiple myeloma patients have an extramedullary disease, with 1-2% demonstrating cutaneous involvement [3-5]. Skin lesions appear commonly as multiple papules, plaques, and/or cutaneous and subcutaneous nodules, with firm consistency, smooth surface, and skin-colored, red, or violaceous [6,7]. Metastatic cutaneous lesions generally appear late in the course of the disease [8]. Nevertheless, they may occur as the first manifestation of the disease [9] or at an earlier stage [7]. Cutaneous multiple myeloma is associated with aggressive biologic behavior and carries very poor prognosis with a median overall survival of approximately eight months as the duration from skin involvement. Despite aggressive modern therapies, including stem cell transplantation, most patients develop progressive disease [5,8,10,11]. Little is known about the mechanism of cutaneous involvement by multiple myeloma, as the medical literature describes only case reports or case series with a limited number of patients. One such case report indicated that the skin-homing of multiple myeloma cells can be related to their high C-C motif chemokine receptor 10 (CCR10) expression at the time of diagnosis, along with the lack of C-X-C chemokine receptor type 4 (CXCR4) surface expression throughout the disease progression. Moreover, high expression of CD44, together with the downregulation of intercellular adhesion molecule-1 (ICAM1)/CD54 and the lack of expression of CD56 by multiple myeloma cells in the progressive disease, with the presence of both antigens during the extramedullary localization can be the mechanisms that drive the escape of plasma cells from bone marrow and their localization into the skin [12].

Multiple myeloma and malignant melanoma

The development of secondary primary malignancies (SPMs) among cancer patients is not an uncommon phenomenon. The National Cancer Institute’s Surveillance, Epidemiology, and End Results Program analyzed its database from 1973 to 2000 and reported that the cumulative incidence of SPMs was nearly 14% at 25 years of follow-up for cancer patients. Myeloma patients had a 6.1% incidence of SPMs at 20 years. However, increased relative risks for acute myelogenous leukemia, chronic myelogenous leukemia, and Kaposi’s sarcoma were noted. The increased risk of developing new malignancy was limited to individuals diagnosed with myeloma at less than 70 years of age [13].

There are limited studies in the literature focusing on the association between multiple myeloma and melanoma. Two studies using population-based data from the Sweden Cancer Registry identified more than 8,000 patients with multiple myeloma and an increased risk for nonmelanoma skin cancer but not for melanoma [14,15]. Another large population-based study of 31,622 patients with multiple myeloma demonstrated no increased risk of melanoma in multiple myeloma patients compared to the general population [16]. However, several studies have shown that the incidence of melanoma increases in several immunocompromised conditions. This difference can be attributed in part to the differential suppression of the cellular constituents of the immune system among various forms of immunosuppression [16]. Cutaneous metastasis in multiple myeloma demonstrates a diverse cytomorphologic spectrum with plasmacytic, plasmablastic, or lymphoplasmacytic features and may show concurrent amyloid deposition or neoplasms such as malignant melanoma and squamous cell carcinoma [8]. Our case is an example of cutaneous multiple myeloma lesions with coexisting malignant melanoma.

Chemotherapy and secondary primary malignancies

There have been significant improvements in the outcome of patients with multiple myeloma in the last two decades with the median overall survival increasing from three to up to eight years [17-19]. In the era of immunomodulatory agents, thalidomide, pomalidomide, and lenalidomide are major maintenance chemotherapy agents for multiple myeloma. However, long-term thalidomide administration is poorly tolerated, mainly due to peripheral neuropathy [20,21]. In contrast, in some studies, lenalidomide, which is a relatively newer agent, has been associated with a manageable side effect profile and significant improvements in progression-free survival and overall survival [22-24]. However, over time, SPMs have been recognized as complications after chemotherapy for other neoplasms such as multiple myeloma, Hodgkin’s lymphoma, and ovarian cancer. Moreover, regardless of the indication, chemotherapy has been shown to be directly responsible for this increased risk due to mechanisms such as direct DNA damage [13]. One such agent which has been studied is lenalidomide which has been associated with the development of SPMs [22-25]. Our patient was also treated with lenalidomide and developed melanoma. Although an association cannot be established with only one case report, this can be an opener for further case reports, series, and larger studies focusing on the association/increased risk of melanoma in patients with multiple myeloma with cutaneous involvement and the relationship with lenalidomide therapy.

## Conclusions

Multiple myeloma is an uncommon type of cancer. Cutaneous metastasis in multiple myeloma is rarer with fewer than 100 cases described in the literature so far. It often indicates a poor prognosis. Though there may be a pathological relationship between the coexistence of malignant melanoma and cutaneous multiple myeloma which is yet to be established, there is a possibility that the use of lenalidomide is associated with the development of secondary malignancy in multiple myeloma patients. We have presented a very rare case of multiple myeloma with cutaneous metastasis who was treated with lenalidomide chemotherapy and developed malignant melanoma of the skin. As the association cannot be established with a single case report, further research should focus on the coexistence of cutaneous multiple myeloma and malignant melanoma.
